# The relationship between self-efficacy and treatment satisfaction among patients with anticoagulant therapy: a cross-sectional study from a developing country

**DOI:** 10.1186/s12959-022-00374-2

**Published:** 2022-04-04

**Authors:** Samah W. Al-Jabi, Amal Abu Dalu, Amer A. Koni, Maher R. Khdour, Adham Abu Taha, Riad Amer, Sa’ed H. Zyoud

**Affiliations:** 1 Department of Clinical and Community Pharmacy, College of Medicine and Health Sciences, An-Najah National University, Nablus, 44839 Palestine; 2 Division of Clinical Pharmacy, Department of Hematology and Oncology, An-Najah National University Hospital, Nablus, 44839 Palestine; 3 Faculty of Pharmacy, Al-Quds University, Abu Deis, Jerusalem, 51000 Palestine; 4 Department of Biomedical Sciences, Faculty of Medicine and Health Sciences, An-Najah National University, Nablus, 44839 Palestine; 5 Department of Pathology, An-Najah National University Hospital, Nablus, 44839 Palestine; 6 Department of Medicine, College of Medicine and Health Sciences, An-Najah National University, Nablus, 44839 Palestine; 7 Department of Hematology and Oncology, An-Najah National University Hospital, Nablus, 44839 Palestine; 8 Poison Control and Drug Information Center (PCDIC), College of Medicine and Health Sciences, An-Najah National University, Nablus, 44839 Palestine; 9 Clinical Research Center, An-Najah National University Hospital, Nablus, 44839 Palestine

**Keywords:** Self-efficacy, SES6C, Treatment satisfaction, Burden, Benefit, ACTS, Anti-coagulant, NOAC

## Abstract

**Background:**

Thromboembolic events are a common complicated health problem. Although anticoagulants have several positive effects on these conditions, they also have several characteristics that strongly affect compliance and satisfaction. The purpose of this investigation is to explore the association between treatment satisfaction and self-efficacy in a sample of patients using anticoagulation therapy and determine the influence of sociodemographic and clinical factors on both aspects.

**Methods:**

This was a cross-sectional exploratory study carried out in Palestine. The Arabic version of the Anti-Coagulant Treatment Satisfaction Scale (ACTS) assessed treatment satisfaction. In addition, the Arabic version of the 6-Item Self-Efficacy for Managing Chronic Diseases (SES6C) was used to assess self-efficacy.

**Results:**

A total of 300 patients using anticoagulants (average age 51.95 and SD 17.98) were included. There is a modest correlation between treatment satisfaction and self-efficacy (r = 0.345; *p* <  0.001). The mean and median self-efficacy scores were 38.41 ± 9.88 and 39.00 (interquartile range: 33.00–46.00), respectively. Overall, patients reported a moderate burden and benefit score. The mean and median of the acting burden were 43.30 ± 10.45, and 43.30 (interquartile range: 36.00 to 51.00), respectively. The results showed that young age, higher education, employment, use of fewer medications, and having fewer diseases were significantly associated with higher self-efficacy behaviors. The results also showed that new oral anti-coagulants (NOACs) had a higher degree of self-efficacy and ACTS benefit scores (41.00 (33.75–47.00), *p* = 0.002; 13.00 (12.00–15.00), *p* <  0.001, respectively), than vitamin k antagonists (VKA).

**Conclusions:**

The results demonstrated a significant relationship between treatment satisfaction and self-efficacy, and certain sociodemographic and clinical characteristics influence both. We found that there is a higher degree of self-efficacy and treatment satisfaction among patients who use NOACs than those who use UFH / VKA. Therefore, patients should be motivated to increase their knowledge about anticoagulant therapy. Healthcare providers should play an active role in educating patients, increasing their self-esteem, and awareness about anticoagulant drugs. Importantly, this study was an explanatory one, and it includes a low proportion of patients with venous thromboembolism. This encourages future research on a large scale of patients, considering the indications of anticoagulant therapy.

## Background

Anticoagulant drugs with different mechanisms of action are indicated for many conditions, such as the prevention of systemic embolism in valvular heart disease, myocardial infarction [1], atrial fibrillation-related stroke [2], pulmonary embolism (PE), deep vein thrombosis (DVT) [3] and cancer-related thrombosis [4]. However, if non-anticoagulated, there is a significant increase in the mortality rate in patients with stroke risk factors [5], morbidity, and negative effects on quality of life [6].

Although anticoagulants have several positive effects on the disease, they also have several characteristics that can strongly affect compliance and satisfaction, inducing dissatisfaction and reducing the patient’s quality of life (e.g., diet and activity restrictions, regular blood tests, bleeding, and/or bruises). These side effects and burdens can lead to poor compliance, and as a result, to failure of treatment failure [3]. In addition, long-term anticoagulant therapy, particularly with vitamin K antagonist (VKA), can be associated with low satisfaction and poor adherence [6].

Patient satisfaction with anticoagulant therapy and his quality of life in clinical practice may affect treatment outcomes [1], as satisfaction can increase compliance and treatment success. Individual-level patient characteristics and preferences for shared decision-making will improve the patient’s treatment experience by achieving treatment satisfaction [7, 8]. Treatment satisfaction was associated with better compliance and persistence, in addition to reducing treatment burden or regimen complexity [9]. This makes satisfaction for an individual patient an important factor in the selection or changing anticoagulant therapy in patients [10]. On the other hand, treatment dissatisfaction can negatively affect patients, such as the quality of the treatment regimen implemented [9].

Another aspect that can affect the success of treatment and its goal is the patient’s self-efficacy. ‘Self-efficacy’ is a term that refers to the confidence one has in the ability to follow the behaviors needed to achieve a desired goal or effect. It is a psychological concept that is used in association with chronic diseases and medication management. There has been a growing interest in the role of self-efficacy as a predictor of the treatment outcome [11]. Self-efficacy helps participants have confidence and skills better to manage their chronic conditions [12, 13]. Self-efficacy has been increasingly recognized as an essential prerequisite for effective self-management of all chronic diseases [14]. Low self-efficacy is one of the main problems that require physical and mental rehabilitation [15]. In this study, we hypothesize that if the patient has the required knowledge and skills, and is confident in managing his chronic condition and anticoagulant medication, he will be more adherent and satisfied with the therapy used.

Thromboembolic events and many cardiovascular diseases are widely treated with anticoagulants, which have great benefit to patients [16] and have many characteristics that can affect patients’ daily lives. Ample of studies have been established to study anticoagulant treatment satisfaction and self-efficacy; however, these studies did not correlate the two concepts with each other [8, 10, 17–28]. Therefore, the purpose of this investigation is to explore the association between treatment satisfaction and self-efficacy in a sample of patients using anticoagulation therapy and determine the influence of sociodemographic and clinical factors on both aspects. To the best of our knowledge, this study is the first of its type in Palestine. Consequently, this study will provide baseline data and information on treatment satisfaction, self-efficacy, and the relationship between the two concepts, in addition to evaluating factors associated with patient self-efficacy and treatment satisfaction with anticoagulant therapy. Furthermore, it will help healthcare providers establish mechanisms that can achieve patient treatment satisfaction and train them to accept good self-efficacy.

## Methods

### Study design

This study was a cross-sectional questionnaire-based exploratory study to measure patient self-efficacy and treatment satisfaction with anticoagulant therapy and to find the relationship between both aspects. This study was conducted in Jerusalem at Al Makassed Tertiary Hospital, from July 2019 to July 2020.

### Study population

The population of this study was patients from hospitals or outpatient clinics who were using anticoagulants.

### Sample size calculation and sampling procedure

The study was carried out in a group of patients who used anticoagulants attending Al Makassed Hospital. Based on the expected population during the research period (*n* = 1000 patients) and a 50% response distribution, the needed sample size was approximately 278 with a confidence level of 95% and a margin of error of 5%. An automated software program, the Raosoft sample size calculator (http://www.raosoft.com/samplesize.html) was used to calculate the required sample size for this study. The intended sample size was increased to 300 individuals to reduce erroneous results and increase the reliability of the study.

### Inclusion and exclusion criteria

The inclusion criteria were patients over 18 years of age using any type of anticoagulant drug, Arab nationality, and who can read or understand Arabic. Exclusion criteria were patients who refused to participate in the study and those previously diagnosed with mental illnesses or severe cerebral vascular disease that affected cognitive ability.

### Data collection and management

This quantitative study used a questionnaire as an instrument to collect data from respondents. This study’s data collection forms were adopted from two different scales, the Anti-Coagulant Treatment Satisfaction Scale (ACTS) and the Self-Efficacy for Managing Chronic Disease 6-Item Scale (SES6C).

The data collection form contains four sections. In the first section, we covered sociodemographic factors, such as age, gender (male, female), residency (city, village, or refugee camp), job, the primary healthcare centers they visit, marital status, body mass index (BMI) and educational level (illiterate, primary, secondary, or university). The second section of the questionnaire consists of questions related to clinical data, including co-morbid conditions, recent major surgery, history of major bleeding, risk of falls, laboratory findings, and type of anticoagulant drug type used, dose, and duration. The third section measures patients’ satisfaction with anticoagulant therapy treatment using the ACTS scale. The ACTS scale is an anticoagulation-related quality of life measurement instrument. This scale is not generic, but it is condition-specific, focusing on the aspects of health-related quality of life specific to anticoagulant medications [1]. The ACTS questionnaire is an instrument used to measure treatment satisfaction with anticoagulant therapy through patient-reported outcomes (PRO). It consists of two parts; the first part includes 12 items that assess the perceived burden of anticoagulant treatment, and the second part consists of 3 items that assess the perceived benefits of anticoagulant treatment. It uses a 5-point intensity scale (from 1 ‘not at all’ to 5 ‘extremely’). Its total burden scores are reversed (higher scores indicate less burden and vice versa) and range from 12 to 60. However, the total scores of the ACTS benefits are directly scored, ranging from 3 to 15 [23, 29, 30]. The questionnaire has been profoundly tested for content validity, question difficulty measures, readability, item/person reliability in many languages [30] (Cano et al., 2012) and specifically Arabic language [6, 31]. We also registered our study with ePROVIDE - Mapi Research Trust and obtained permission for the use of ACTS use (ID: 42874). The fourth section is used to measure the self-efficacy in anticoagulant therapy using the SES6C. The SES6C is a valid and reliable instrument for assessing the self-efficacy in the management of chronic diseases. It is a self-reporting instrument that can be used to assess a degree of confidence of the patient who is suffering from chronic disease in trying to manage their disease [32]. It is a 6-item scale with a visual analogue, ranging from 1 (not at all confident) to 10 (totally confident). This scale covers several common domains in many chronic diseases, role function, emotional functions, symptom control, and communication with physicians. It is an important and reliable instrument for assessing self-efficacy in managing different chronic diseases. A higher score indicates greater confidence in the management of the patient’s chronic disease.

A qualified pharmacist collected the required data and administered the questionnaire. The pharmacist met the patient in a clinic or pharmacy. Patients were advised that the questionnaire would take approximately 20 min to complete.

### Ethical approval

All aspects of the study protocol, including access to and use of patient clinical information, were approved by the *Institutional Review Board (IRB) of An-Najah National University* and local health authorities prior to the initiation of this study. In addition, a verbal consent form was obtained from each patient.

### Pilot study

A pilot study of 30 participants has been conducted to test the tool, ensure the availability of the required data, estimate the time, and modify the data collection form, as appropriate. Patients who participated in the pilot study were not included in the final analysis.

### Statistical analysis

Data were entered and analyzed using the Statistical Package for Social Sciences program version 15 (SPSS). Data were expressed as means ± SD for continuous variables and as frequencies (percentages) for categorical variables. Variables that are not normally distributed are expressed as medians (lower-upper quartiles). The normality of the variables was tested using the Kolmogorov–Smirnov test and found that the data were not normally distributed. Therefore, non-parametric tests of univariate analysis were used to test for differences in medians between categories. Indeed, the Mann-Whitney test was used to compare the two groups of patients’ characteristics on the dependent variables (self-efficacy, burden, and benefit scores), while the Kruskal–Wallis was applied to compare three or more groups. The significance level was established at a *p*-value of < 0.05.

## Results

### Sociodemographic and clinical data

Our study was carried out on 323 patients with a response rate of 92.88%. Table 1 indicates that most of our patients are women (61.7%), nearly half of them (54.3%) are middle-aged (30–60 years), and 35% are over 60 years old. Additionally, 55.7% of our patients had a secondary level of education or less (18% secondary, 17.0% primary, 14.0% elementary, and 6.7% were illiterate), and 41.3% had a high level of education. Of 300 patients, 64.7% were employed, 53.3% had a monthly income between 2000 and 5000 NIS, and 36.0% had a monthly income of more than 5000 NIS. Depending on the location, 57.3% of our patients were urban, 28.3% Rural, and 12.0% lived in camps. Almost half of the patients (48.7%) had three or more diseases. The reason for the anticoagulation therapy was AF 21%, DVT or PE 9.7%, as prophylaxis during hospital admission 24%, and other indications 45.3%. The mean number of medications used by our patients was 4.2. This number was used to categorize this variable. Therefore, 58.7% of the participants used four or less than four medications.

**Table 1:** Sociodemographic and clinical characteristics of the study sample

Sociodemographic Variables	Frequency (%) ***N*** = 300
**Gender**	
Male	115 (38.3)
Female	185 (61.7)
**Age**	
Less than 30	31 (10.3)
30–60	163 (54.3)
More than 60	106 (35.3)
**BMI**	
Normal weight (18.5–24.9)	65 (21.7)
Overweight (25–29.9)	136 (45.3)
Obese > 30	93 (31.0)
**Educational status**^**a**^	
Illiterate	20 (6.7)
Elementary	42 (14.0)
Primary	51 (17.0)
Secondary	54 (18.0)
University	124 (41.3)
**Marital status**^**a**^	
Married	229(76.3)
Unmarried (single, divorced, widowed)	67(22.3)
**Income/month (NIS)**^**a**^	
Less than 2000	23 (7.7)
2000–5000	160(53.3)
More than 5000	108(36.0)
**Employment status**^**a**^	
Employed	194(64.7)
Unemployed	100(33.3)
**Locality**^**a**^	
Camp	36(12.0)
Rural	85(28.3)
Urban	172(57.3)
**Chronic co-morbid disease**^**a**^	
No co-morbid disease (pregnant)	42 (14.0)
One Disease	44(14.7)
Two diseases	63(21.0)
Three diseases or more	146(48.7)
**Indications for anticoagulant**	
Atrial fibrillation	63 (21.0)
Deep vein thrombosis or Pulmonary embolism	29 (9.7)
Prophylaxis^**b**^	72 (24.0)
Other indications	136 (45.3)
**Chronic medications**	
**≤ 4**	176(58.7)
**> 4**	124(41.3)

**Abbreviations;** *NIS* New Israeli Shekel (0.29 US Dollar), *BMI* Body mass index. ^**a**^Some data were missing

^**b**^Patients that received prophylactic doses of unfractionated heparin during hospital admission

### Self-efficacy score according to sociodemographic variables

The mean and median self-efficacy score was 38.41 ± 9.88, and 39.00 (interquartile range: 33.00–46.00), respectively. A significant difference in self-efficacy scores was found between participants and their age (*p* <  0.001), educational level (*p* <  0.001), employment status (*p* = 0.049), comorbidities (*p* = 0.034) and chronic medications (*p* = 0.029), and anticoagulant drug (*p* = 0.002). The median of self-efficacy scores in participants younger than 30 years of age was 47.00 (41.00–50.00) compared to 35.00 (30.00–42.00) for those who were older than 60. Furthermore, illiterate participants showed the lowest self-efficacy scores among the education status category, with a median score of 29.50 (21.75–41.5), while the median self-efficacy scores for participants with a university education level was 41.50 (34.00–47.00). For participants who had only one chronic disease, the median self-efficacy score was 42.00 (34.00–49.00), the median reduced with participants having two chronic diseases 40.00 (33.00–47.00), and the lower score was for participants having three or more chronic diseases 37.00 (30.75–44.00). Furthermore, the median score for participants taking four or fewer medications was 41.00 (33.25–47.0), higher than participants taking more than four medications 37.00 (32.00–44.00). The self-efficacy score according to the anticoagulant used was 41.00 (33.75–47.00) for NOACs, 40.00 (35.00–46.00) for UFH, and 35.00 (30.25–42.00) for warfarin users. Additionally, Patients with DVT or PE scored higher on the self-efficacy scale (*p* = 0.001).

No significant differences were observed between participants according to their gender, weight, income, location, and marital state (Table 2).

**Table 2:** Total self-efficacy score by socio-demographic and clinical variables

Variable	Frequency % N(300)	Median (Q1–Q3)	Mean ± SD	Mean rank	P-value
**Age category (years)**					**< 0.001** ^**b**^
< 30	31 (10.3)	47.00(41.00–50.00)	44.32 ± 9.09	205.84	
30–60	163 (54.3)	41.00(35.00–46.00)	39.36 ± 9.38	158.72	
> 60	106 (35.3)	35.00(30.00–42.00)	35.23 ± 9.80	121.68	
**Gender**					0.311 ^a^
Male	115 (38.3)	40.00(33.00–47.00)	39.09 ± 10.46	156.93	
Female	185 (61.7)	39.00(32.00–45.00)	37.99 ± 9.52	146.50	
**BMI**^**a**^					0.146^**b**^
Normal	65 (21.7)	41.00(39.00–49.00)	40.13 ± 9.51	163.11	
Overweight	136 (45.3)	39.00(34.00–45.00)	38.35 ± 9.05	147.75	
Obese	93 (31.0)	38.00(31.00–44.00)	37.09 ± 9.97	136.19	
**Education**					**< 0.001** ^**b**^
Illiterate	20 (6.7)	29.50(21.75–41.50)	30.70 ± 11.37	90.18	
Elementary	43 (14.3)	35.00(28.00–41.00)	36.14 ± 9.42	123.37	
Primary	52 (17.3)	37.00(31.00–42.00)	36.45 ± 9.77	130.83	
Secondary	54 (18.0)	41.00(36.75–48.00)	40.65 ± 9.91	167.40	
University	124 (41.3)	41.50(34.00–47.00)	39.85 ± 9.18	159.59	
**Income (month)**					0.432^**b**^
Less than 2000 NIS	23 (7.7)	41.00(31.00–45.00)	37.61 ± 11.70	145.93	
2000–5000 NIS	160(53.3)	39.00(31.00–45.00)	38.00 ± 9.66	140.54	
More than 5000 NIS	108(36.0)	41.00 (35.00–46.00)	39.31 ± 10.07	154.10	
**locality**					0.675 ^**b**^
Camp	36 (12.0)	38.50(31.25–45.75)	37.53 ± 9.46	138.42	
Rural	85(28.3)	39.00(31.00–44.00)	38.11 ± 9.51	143.67	
Urban	172(57.3)	39.00(34.00–46.75)	38.76 ± 10.18	150.44	
**Marital status**					0.103^a^
Married	229(76.3)	12.00(11.00–14.00)	39.00 ± 9.52	152.59	
Unmarried (single, divorced, widowed)	67(22.3)	11.00(10.00–13.00)	36.25 ± 10.97	133.50	
**Employment status**					**0.049** ^**a**^
Employed	194(64.7)	42.00(34.00–48.00)	40.04 ± 10.26	161.07	
Unemployed	100(33.3)	38.00(32.00–44.25)	37.82 ± 9.24	140.51	
**Chronic co-morbid disease**					**0.034** ^**b**^
No co-morbid disease	42 (14.0)	42.00(35.00–47.00)	40.29 ± 8.89	166.74	
One disease	44(14.7)	42.00(34.00–49.00)	40.30 ± 10.56	164.71	
Two diseases	63(21.0)	40.00 (33.00–47.00)	39.43 ± 8.89	156.02	
Three diseases or more	146(48.7)	37.00(30.75–44.00)	36.76 ± 10.32	133.22	
**Indications for anticoagulant**					**0.001** ^**b**^
Atrial fibrillation	63 (21.0)	36.00 (27.00–41.00)	34.02 ± 10.51	115.01	
Deep vein thrombosis or Pulmonary embolism	29 (9.7)	43.00 (34.51–49.50)	42.76 ± 7.79	187.07	
Prophylaxis^c^	72 (24.0)	39.50 (35.00–46.75)	38.63 ± 8.83	152.99	
Other indications	136 (45.3)	41.00 (33.00–47.00)	39.40 ± 9.90	157.82	
**Chronic medications**					**0.029** ^**a**^
≤ 4	176(58.7)	41.00(33.25–47.00)	39.32 ± 9.95	159.76	
> 4	124(41.3)	37.00(32.00–44.00)	37.11 ± 9.68	137.49	
**Anticoagulant drug**					**0.002** ^**b**^
UHF	87(29)	40.00(35.00–46.00)	39.02 ± 8.45	154.05	
Vit-K dependent	134(44.7)	35.00(30.25–42.00)	35.18 ± 9.74	119.38	
NOACs	75(25)	41.00(33.75–47.00)	39.00 ± 8.20	161.20	

^a^ The statistical significance of the differences was calculated using the Mann-Whitney U test

^b^ The statistical significance of the differences was calculated using the Kruskal-Wallis test

Bold P-value indicates a significant difference

^c^Patients that received prophylactic doses of unfractionated heparin during hospital admission

NIS: New Israeli Shekel (0.29 US Dollar)

### ACTS burden score according to sociodemographic variables

The mean and median of the acting burden were 43.30 ± 10.45, and 43.30(interquartile range: 36.00 to 51.00), respectively. According to the gender of the participant, the ACTS burden score for the male participants was 46.00 (38.00–52.00), and the female 42.00 (35.00–49.00) (*p* = 0.006). The median ACTS burden scores significantly increase with increasing monthly income (*p* = 0.001), 40.00 (31.00–46.00) was for a monthly income of less than 2000 NIS, 42.00 (35.00–50.00) for 2000–5000 NIS, and 45.00 (40.00–53.00) for a monthly income of more than 5000 NIS. Married participants had a higher ACTS burden score 44.00 (37.00–51.00) than unmarried (single, divorced and widowed) 41.00 (31.00–49.00), (*p* <  0.001). According to the indications, no significant differences were found in the burden scores. Depending on the type of anticoagulant used, there were no significant differences in the median burden score of treatment satisfaction between UFH, VKA and NOACs (Table 3).

**Table 3:** Total burden scores by socio-demographic and clinical variables

Variable	Frequency % N(300)	Median (Q1–Q3)	Mean ± SD	Mean rank	P-value
**Age category (years)**					0.651 ^**b**^
< 30	31 (10.3)	42.00(34.00–52.00)	41.68 ± 11.02	141.21	
30–60	163 (54.3)	43.00(37.00–51.00)	43.99 ± 10.77	154.47	
> 60	106 (35.3)	45.00(36.00–50.00)	42.73 ± 91.78	147.11	
**Gender**					**0.006** ^**a**^
Male	115 (38.3)	46.00(38.00–52.00)	45.01 ± 9.88	168.03	
Female	185 (61.7)	42.00(35.00–49.00)	42.25 ± 10.68	139.60	
**BMI**^**a**^					0.702 ^**b**^
Normal	65 (21.7)	42.00(35.00–50.00)	42.89 ± 9.89	140.45	
Overweight	136 (45.3)	44.00(36.25–51.75)	43.44 ± 10.15	151.19	
Obese	93 (31.0)	44.00(37.00–50.50)	43.55 ± 11.48	147.04	
**Education**					0.160 ^**b**^
Illiterate	20 (6.7)	39.00(29.25–49.50)	39.60 ± 11.17	118.63	
Elementary	43 (14.3)	46.00(36.75–50.25)	43.31 ± 9.19	149.86	
Primary	52 (17.3)	42.00(35.00–49.00)	41.06 ± 10.13	131.38	
Secondary	54 (18.0)	45.00(38.75–54.00)	45.67 ± 9.60	165.19	
University	124 (41.3)	43.00(36.00–50.75)	43.80 ± 11.25	146.76	
**Income (month)**					**0.001**^**b**^
Less than 2000 NIS	23 (7.7)	40.00(31.00–46.00)	38.60 ± 8.74	108.11	
2000–5000 NIS	160(53.3)	42.00(35.00–50.00)	41.93 ± 9.44	136.66	
More than 5000 NIS	108(36.0)	45.00(40.00–53.00)	46.06 ± 11.10	167.91	
**locality**					0.123 ^**b**^
Camp	36(12.0)	41.00(35.00–48.50)	41.47 ± 8.55	131.01	
Rural	85(28.3)	40.00(34.00–50.00)	41.83 ± 11.09	136.84	
Urban	172(57.3)	44.00(37.25–51.00)	49.17 ± 10.13	155.37	
**Marital status**					**0.005**^**a**^
Married	229(76.3)	44.00(37.00–51.00)	44.29 ± 10.26		
Unmarried (single, divorced, widowed)	67(22.3)	41.00(31.00–49.00)	39.86 ± 10.32		
**Employment status**					0.114 ^a^
Employed	194(64.7)	42.00(35.75–50.25)	44.76 ± 11.55	158.40	
Unemployed	100(33.3)	45.00(36.25–52.00)	42.61 ± 9.88	141.88	
**Chronic co-morbid disease**					0.696 ^**b**^
No co-morbid disease	42 (14.0)	44.00(38.00–52.00)	44.24 ± 8.25	156.31	
One disease	44(14.7)	45.00(35.00–53.00)	44.67 ± 11.45	156.79	
Two diseases	63(21.0)	42.00(36.00–51.00)	42.35 ± 9.26	141.24	
Three diseases or more	146(48.7)	44.00(36.00–50.00)	43.41 ± 11.05	144.93	
**Indications for anticoagulant**					0.366 ^**b**^
Atrial fibrillation	63 (21.0)	43.00 (37.00–50.00)	42.54 ± 8.60	145.65	
Deep vein thrombosis or Pulmonary embolism	29 (9.7)	41.00 (34.00–46.50)	41.00 ± 8.41	126.03	
Prophylaxis ^c^	72 (24.0)	44.00 (37.00–52.00)	43.57 ± 10.16	154.79	
Other indications	136 (45.3)	45.00 (36.00–51.00)	44.01 ± 11.71	155.69	
**Chronic medications**					0.932 ^a^
**≤4**	176(58.7)	43.00(36.00–51.00)	43.43 ± 11.05	150.86	
**> 4**	124(41.3)	43.00 (36.00–50.00)	43.14 ± 9.57	149.99	
**Anticoagulant drug**					
UHF	87(29)	42.00(35.00–51.00)	42.75 ± 9.63	143.83	0.596 ^**b**^
Vit-K dependent	134(44.7)	43.00 (36.00–51.00)	41.65 ± 11.06	143.90	
NOACs	75(25)	44.00 (36.75–57.00)	44.12 ± 11.43	154.06	

^a^ The statistical significance of differences was calculated using the Mann-Whitney U test

^b^ The statistical significance of the differences was calculated using the Kruskal-Wallis test

Bold P-value indicates a significant difference

^c^Patients that received prophylactic doses of unfractionated heparin during hospital admission

NIS: New Israeli Shekel (0.29 US Dollar)

### ACTS benefit score according to sociodemographic variables

The mean and median of the total ACTS benefit total score was 11.84 ± 2.17, and 12.00 (interquartile range 11.00–13.75), respectively. The median of the ACTS benefit scores in participants under 30 years old was 15.00 (14.00–15.00), middle age (30–60) was 12.00 (11.00–14.00), and older than 60 was 11.00 (9.00–12.00). Depending on the gender of the participants, the median ACTS benefit score for male was 12.00 (10.00–12.00), and female 12.00 (11.00–14.00). Illiterate participants had the lowest median ACTS benefit score of 9.50 (9.00–11.00), elementary school level was 11.00 (9.00–12.00), primary school was 12.00 (10.00–13.00), secondary school was 12.00 (11.00–14.00), and university 12.00 (11.00–15.00). Furthermore, participants with one or two diseases had a median score was 12.00 (10.00–14.00), while participants with three or more have a lower score of 11.00 (10.00–12.00). The ACTS benefit score was 12.00 (11.00–15.00) for participants using four or less and 11.00 (10.00–12.00) for participants using more than four medications. The median benefit score was significantly higher for those who received prophylactic doses of enoxaparin during hospital admission (*p* <  0.001). Finally, the median of the ACTS benefit score according to the anticoagulants used was 11.00 (10.00–12.00) for enoxaparin, 11.50 (10.00–13.00) for warfarin, and 13.00 (12.00–15.00) for NOAC users. Table 4 shows a significant acting benefit score based on the participant’s age (*p* <  0.001), gender (*p* = 0.015), BMI (*p* = 0.003), educational level (*p* <  0.001), marital status (*p* = 0.003), comorbidities (*p* = 0.018), chronic medications (*p* < 0.001), indication (*p* < 0.001), and anticoagulant drug (*p* < 0.001).

**Table 4:** Benefits total by score sociodemographic and clinical variables

Variable	Frequency % N(300)	Median (Q1–Q3)	Mean ± SD	Mean rank	***P*** value
**Age category (years)**					**< 0.001** ^**b**^
< 30	31 (10.3)	15.00(14.00–15.00)	14.55 ± 0.89	255.40	
30–60	163 (54.3)	12.00(11.00–14.00)	12.08 ± 1.97	159.84	
> 60	106 (35.3)	11.00(9.00–12.00)	10.69 ± 1.93	105.45	
**Gender**					**0.015** ^**a**^
Male	115 (38.3)	12.00(10.00–12.00)	11.47 ± 1.98	133.34	
Female	185 (61.7)	12.00(11.00–14.00)	12.07 ± 2.26	159.92	
**BMI**^**a**^					**0.003**^**b**^
Normal	65 (21.7)	12.00(11.00–15.00)	12.63 ± 2.08	178.75	
Overweight	136 (45.3)	12.00(10.00–13.00)	11.57 ± 2.30	139.13	
Obese	93 (31.0)	12.00(10.00–13.00)	11.58 ± 1.94	137.90	
**Education**					**< 0.001** ^**b**^
Illiterate	20 (6.7)	9.50(9.00–11.00)	10.05 ± 1.57	75.45	
Elementary	43 (14.3)	11.00(9.00–12.00)	10.76 ± 1.76	101.92	
Primary	52 (17.3)	12.00(10.00–13.00)	11.73 ± 2.15	145.35	
Secondary	54 (18.0)	12.00(11.00–14.00)	12.19 ± 2.13	159.62	
University	124 (41.3)	12.00(11.00–15.00)	12.38 ± 2.15	166.65	
**Income (month)**					0.641 ^**b**^
Less than 2000 NIS	23 (7.7)	12.00(11.00–15.00)	12.17 ± 2.61	160.98	
2000–5000 NIS	160(53.3)	12.00(11.00–14.00)	11.82 ± 2.20	145.83	
More than 5000 NIS	108(36.0)	12.00(10.00–13.00)	11.75 ± 2.09	143.06	
**locality**					0.258 ^**b**^
Camp	36 (12.0)	12.00(11.00–14.75)	12.25 ± 2.08	161.86	
Rural	85(28.3)	12.00(11.00–15.00)	12.02 ± 2.30	153.71	
Urban	172(57.3)	12.00(10.00–13.00)	11.66 ± 2.14	140.58	
**Marital status**					**0.003** ^**b**^
Married	229(76.3)	12.00(11.00–14.00)	12.01 ± 2.14	127.51	
Unmarried (single, divorced, widowed)	67(22.3)	11.00(10.00–13.00)	11.37 ± 2.14	154.64	
**Employment status**					0.188 ^a^
Employed	194(64.7)	12.00(10.00–13.25)	12.10 ± 1.80	156.46	
Unemployed	100(33.3)	12.00(11.00–14.00)	11.73 ± 2.32	142.88	
**Chronic co-morbid disease**					**0.018**^**b**^
No co-morbid disease	42 (14.0)	14.00(12.00–15.00)	12.14 ± 1.96	216.07	
One disease	44(14.7)	12.00(10.00–14.00)	11.99 ± 2.10	152.50	
Two diseases	63(21.0)	12.00(10.00–14.00)	12.05 ± 2.06	154.00	
Three diseases or more	146(48.7)	11.00(10.00–12.00)	11.20 ± 2.05	123.50	
**Indications for anticoagulant**					**< 0.001** ^**b**^
Atrial fibrillation	63 (21.0)	11.00 (9.00–12.00)	10.84 ± 1.90	112.70	
Deep vein thrombosis or Pulmonary embolism	29 (9.7)	12.00 (10.50–15.00)	12.31 ± 2.58	167.93	
Prophylaxis ^c^	72 (24.0)	13.00 (12.00–15.00)	13.11 ± 1.87	203.99	
Other indications	136 (45.3)	11.00 (10.00–12.75)	11.53 ± 2.03	135.98	
**Chronic medications**					**< 0.001** ^**a**^
≤4	176(58.7)	12.00(11.00–15.00)	12.25 ± 2.23	167.30	
> 4	124(41.3)	11.00(10.00–12.00)	11.26 ± 1.96	126.65	
**Anticoagulant drug**					**< 0.001** ^**a**^
UHF	87(29)	11.00(10.00–12.00)	11.04 ± 1.74	113.63	
Vit-K dependent	134(44.7)	11.50(10.00–13.00)	11.52 ± 2.22	135.54	
NOACs	75(25)	13.00(12.00–15.00)	13.07 ± 1.89	198.53	

^a^ The statistical significance of the differences was calculated using the Mann-Whitney U test

^b^ The statistical significance of the differences was calculated using the Kruskal-Wallis test

Bold P-value indicates significant differences.^c^ Patients that received prophylactic doses of unfractionated heparin during hospital admission

NIS: New Israeli Shekel (0.29 US Dollar)

### Correlations between treatment satisfaction and self-efficacy

The values of the Spearman correlation coefficient between the total burden, benefits, and overall satisfaction score with the self-efficacy score were 0.325, 0.171 and 0.345, respectively. The results of this study indicated a significant modest positive correlation between all satisfaction domains (burdens (*p* < 0.001), benefits (*p* = 0.003) and overall satisfaction (*p* < 0.001)) and self-efficacy scores (Table 5).

**Table 5:** Correlation coefficient between Treatment Satisfaction and Self-Efficacy

Satisfaction Domain	Spearman’s Rho	Self-Efficacy
**Burdens**	Correlation coefficient	0.325
	Significance (2-tailed)	**< 0.001**
**Benefits**	Correlation coefficient	0.171
	Significance (2-tailed)	**0.003**
**Overall satisfaction**	Correlation coefficient	0.345
	Significance (2-tailed)	**< 0.001**

## Discussion

Our study was carried out at Al Makassed hospital, which is located in Jerusalem city in Palestine, in patients who use anticoagulant drugs prophylactically or as a treatment for different medical conditions. This study was one of the first conducted in Palestine to examine whether there is a significant relationship between patient treatment satisfaction using the ACTS scale and self-efficacy using the SES6C scale and to determine the factors that affected both scales.

Our study demonstrated a mean self-efficacy score (SES6C score) of 38.41 and revealed that older patients, less educated, and unemployed patients had a significantly lower self-efficacy score. This necessitates appropriate education and counseling for these patients about their anticoagulant medication. The median score for those who were using Vit-K drugs was 41.00 out of 60. In addition, clinical factors, such as using Vit-K drugs, having a higher number of chronic diseases, and polypharmacy, significantly lower self-efficacy. Especially for Vit-K drugs, special attention and self-management strategies are necessary because these medications have a greater risk of major bleeding [33] and therapeutic failure [34, 35]. Since positive correlations were found between self-efficacy and treatment satisfaction, consequently affecting patient adherence to anticoagulant drugs and success of treatment. Health care providers (mainly physicians and pharmacists) should pay more attention to this issue and consider these factors in the treatment and monitoring plan. Several previous analyzes ensured that self-management in patients using anticoagulants was applicable and positively affected treatment-related quality of life compared to routine care [36–39]. Self-managing patients may be more aware of any change in their INR due to their ability to measure it and take an adaptive action, having higher knowledge, and greater self-efficacy. This reinforces the evidence demonstrating that warfarin therapy self-management is an effective and safe alternative to routine management [40–43]. However, the impact of this strategy on the risk of bleeding and thrombosis should be tested.

In recent years, greater interest has been shown in the study of treatment satisfaction. A Spanish study aimed to evaluate the satisfaction with treatment of nonvalvular atrial fibrillation in anticoagulant patients on anticoagulants in Spain in 2018 [23]. Regarding anticoagulation satisfaction, the ACTS burden scale was 49.69 ± 9.45 and the benefits scale was 11.35 ± 2.61. Higher scores denote a lower burden and higher perceived benefit for both scales, and consequently, greater satisfaction with treatment. Our patients reported a mean burden score of 43.30 out of 60 and a benefit score of 11.84 out of 15. However, our patients reported lower burden scores than other populations [6, 23], which means a higher burden of this therapy.

The burden mean score revealed that the male participants had a higher degree of satisfaction degree than females, which corresponds to a study carried out in Spain [23] and another study, which found that the more burdened and less satisfied participants who used warfarin were more likely to be women [44]. According to our results, young age and women were more perceived to benefit from anticoagulant therapy, but burden scores were not significantly different between age categories. A study conducted in California in patients with venous thromboembolism found that women of younger age were associated with a higher perceived anticoagulant burden [22]. Two Spanish studies, one that dealt with AF patient satisfaction with NOAC [28] and the second specified nonvalvular AF patients’ satisfaction with VKA [21], showed that elderly patients were less burdened with anticoagulant treatment. Furthermore, the benefit score was reported to be significantly reduced in patients with low education level. A Sudanese cross-sectional study published in 2017 [20], evaluated patient satisfaction with oral anticoagulant therapies and their adherence to them and identified the predictors of the two study domains. Approximately half of the participants (50.5%) were satisfied with anticoagulant treatment. The study concludes that a multidisciplinary effort from healthcare providers is needed to ensure health education, in addition to continuously motivating patients continuously in order to increase treatment success, especially among patients with a low educational level.

Patient satisfaction with each anticoagulant drug should be determined among different indications, in order to select a suitable therapy with a low treatment burden on patients that improves clinical outcomes and quality of life. An important finding was that patients who used NOAC had higher scores of both parts of treatment satisfaction; burden and benefit than patients on unfractionated heparin or vitamin k-dependent anticoagulants. However, the significant differences were only for benefit (*p* < 0.001) score only. A substudy of a multicenter research (SAKURA AF registry) aimed to compare the satisfaction of NOAC users and warfarin users in patients with AF using the ACTS scale [26]. The burden scores were significantly higher (54.5 ± 6.3 versus 52.7 ± 6.9, *P* < 0.0001) among NOAC users than among warfarin users, but the baseline benefit scores among NOAC users tended to be lower (9.8 ± 3.1 versus 10.1 ± 3.2, *P* = 0.0513), which is contrary to our results. The reduced burden of NOACs and constant patient education about the benefit of stroke prophylaxis will lead to greater adherence of patients to their treatment plan and therefore have a positive effect on clinical outcomes. SAFARI, a French observational study published in 2016 [24], aimed to investigate patient-reported satisfaction with treatment with rivaroxaban to prevent stroke in patients with AF. A total of 405 nonvalvular atrial fibrillation patients who had been previously treated with warfarin switched to rivaroxaban. Patient satisfaction improved after 3 months and persisted for 6 months. The same observation was found in a Japanese study AGAIN [10] and others [18, 25]. The AGAIN study showed an improvement in treatment satisfaction after switching from warfarin to apixaban in patients with nonvalvular AF (NVAF) after switching from warfarin to apixaban from warfarin. Furthermore, the effect of NOAC therapy in improving treatment satisfaction compared to other anticoagulants was found in the treatment of patients with acute symptomatic DVT and PE [17, 27]. According to the indications, patients who received prophylactic doses of enoxaparin during hospital admission had higher benefit scores, indicating higher treatment satisfaction. This could be explained by the ease of administration, as the prophylactic dose may have been administered by a nurse, this would be different from patients undergoing self-administration.

Increasing patient satisfaction is a role that healthcare providers should be carried out through educating patients about the use of medications, their importance, side effects, and other drug-related factors. Educating the anticoagulated patient is often neglected because it is time-consuming, but pharmacists can play an essential role in this education strategy [45]; pharmacists (according to the National Patient Safety Agency) are in a prime position to provide good counseling about the anticoagulant and disease status [46], since patients forget 40–80% of the information that their doctors give them by their physicians [45].

### Strengths and limitations

To our knowledge, this study was one of the first to investigate the relationship between self-efficacy and treatment satisfaction among patients using anticoagulants in Palestine. On the other hand, this study has a number of limitations. First, this is a cross-sectional study; therefore, it is difficult to prove causal relationships between the scales that have been used and their associated factors. Second, this study did not explore other potential factors that may affect self-care/self-efficacy, such as the duration of the disease, smoking status, economic status (as NOACs were not covered by health insurance), INR monitoring practice, and duration of anticoagulation use. Third, the interviewer’s bias in the results may have been introduced, since the data was collected via a face-to-face interview. Although the study included patients with different anticoagulant medications, bias related to the dosage form of the medication used (oral, intravenous, or subcutaneous) may be introduced as the nature of administration is different and its use is likely to be restricted to a distinct patient subset. Furthermore, there is a potential bias related to the indications since these relate to different patients, as for age, sex, and comorbidity. Finally, the sample size and the use of a single center to recruit patients are considered limiting factors in this study.

## Conclusions

We found a relationship between patients’ self-efficacy on anticoagulant drugs and treatment satisfaction. This correlation is expressed by the low correlation coefficient. A notable finding is that there is a higher degree of self-efficacy and treatment satisfaction among patients using NOACs than among those who use UFH/VKAs. Furthermore, certain factors were found to be significantly associated with self-efficacy and treatment satisfaction. These factors should be considered when creating a treatment plan.

The study’s findings have certain implications for future practice, including the following; we advocate policymakers and healthcare providers to make the necessary changes to improve healthcare services and facilities for all patients, especially outside the cities. In addition, we recommend conducting specialized therapy and training sessions on anticoagulant therapy. Patients should be motivated to increase their knowledge of anticoagulant therapy, which can be achieved through customized health promotions and counseling programs and tailored health promotions. Healthcare providers should play an active role in educating patients, increasing their self-esteem, and awareness about anticoagulant drugs. Importantly, this study was an explanatory one, and it includes a low proportion of patients with venous thromboembolism. This encourages future research on a large scale of patients, considering the indications of anticoagulant therapy.

## Data Availability

All data used in this study are available from the corresponding author upon request.

## Abbreviations

ACTS: Anti-Clot Treatment Scale
AF: Atrial Fibrillation
BMI: Body Mass Index
CVD: Cardiovascular Disease
DVT: Deep Vein Thrombosis
HF: Heart Failure
IHD: Ischemic Heart Disease
IRB: Institutional Review Boards
NIS: New Israeli Shekel
NOACS: New Oral Anticoagulants
PE: Pulmonary Embolism
PRO: Patient Reported Outcome
SES6C: Self-Efficacy for Managing Chronic Disease 6-Item Scale
SPSS: Statistical package for Social sciences
UFH: Unfractionated Heparin
VKAs: Vitamin K Antagonists
